# Transcription factors NRF2 and HSF1 have opposing functions in autophagy

**DOI:** 10.1038/s41598-017-11262-5

**Published:** 2017-09-08

**Authors:** Sharadha Dayalan Naidu, Dina Dikovskaya, Egle Gaurilcikaite, Elena V. Knatko, Zachary R. Healy, Hema Mohan, Glenn Koh, Axel Laurell, Graeme Ball, David Olagnier, Laureano de la Vega, Ian G. Ganley, Paul Talalay, Albena T. Dinkova-Kostova

**Affiliations:** 1 Jacqui Wood Cancer Centre, Division of Cancer Research, School of Medicine, University of Dundee, Dundee, DD1 9SY Scotland UK; 2 Department of Pharmacology and Molecular Sciences, Johns Hopkins University School of Medicine, Baltimore, MD 21205 USA; 3 Dundee Imaging Facility, University of Dundee, Dundee, DD1 9SY, Scotland, UK; 4 Lady Davis Institute-Jewish General Hospital, McGill University, Division of Experimental Medicine, Montreal, QC Canada; 5 The Medical Research Council (MRC) Protein Phosphorylation and Ubiquitylation Unit, School of Life Sciences, University of Dundee, Dundee DD1 5EH, Scotland, UK; 6 Present Address: Wolfson Centre for Age-Related Diseases, King’s College London, London, UK; 7 Present Address: Department of Pulmonary and Critical Care Medicine and Department of Internal Medicine, Duke University Hospital, Durham, NC 27705 USA; 8 Present Address: Department of Biomedicine, Aarhus Research Center for Innate Immunology, Aarhus University, Aarhus, 8000 Denmark

## Abstract

Autophagy plays a critical role in the maintenance of cellular homeostasis by degrading proteins, lipids and organelles. Autophagy is activated in response to stress, but its regulation in the context of other stress response pathways, such as those mediated by heat shock factor 1 (HSF1) and nuclear factor-erythroid 2 p45-related factor 2 (NRF2), is not well understood. We found that the Michael acceptor *bis*(2-hydoxybenzylidene)acetone (HBB2), a dual activator of NRF2 and HSF1, protects against the development of UV irradiation-mediated cutaneous squamous cell carcinoma in mice. We further show that HBB2 is an inducer of autophagy. In cells, HBB2 increases the levels of the autophagy-cargo protein p62/sequestosome 1, and the lipidated form of microtubule-associated protein light chain 3 isoform B. Activation of autophagy by HBB2 is impaired in NRF2-deficient cells, which have reduced autophagic flux and low basal and induced levels of p62. Conversely, HSF1-deficient cells have increased autophagic flux under both basal as well as HBB2-induced conditions, accompanied by increased p62 levels. Our findings suggest that NRF2 and HSF1 have opposing roles during autophagy, and illustrate the existence of tight mechanistic links between the cellular stress responses.

## Introduction

The ability to respond to stress in a dynamic environment is essential for the adaptation and survival of all living organisms. The phase 2 response and the heat shock response represent two essential components of the mammalian stress response and are controlled by transcription factors nuclear factor-erythroid 2 p45-related factor 2 (NRF2) and heat shock factor 1 (HSF1), respectively^1–4^. Although the phase 2 response and the heat shock response are not dependent on each other, mounting evidence suggests that they engage in crosstalk^5^.

Autophagy plays a critical role in the maintenance of cellular homeostasis by degrading proteins, lipids and organelles^6, 7^. Modulation of autophagy is regarded as means for the development of strategies for disease prevention and treatment^8^. Several reports have described links between the heat shock response and autophagy, as well as between the phase 2 response and autophagy. The autophagy-cargo protein p62, also known as sequestosome 1 (SQSTM1) is regulated by oxidative stress and is a transcriptional target of NRF2^9–12^. Furthermore, p62 when phosphorylated, competes with NRF2 for binding to its main negative regulator, Kelch-like ECH-associated protein 1 (KEAP1)^13, 14^, thereby leading to enhanced transcription of NRF2-dependent genes, and creating a positive feedback regulatory loop for its own gene expression. In addition to p62, NRF2 regulates the expression of a number of genes encoding proteins with critical functions in autophagy, including ULK1 and ATG5^15^. Very recently, it was shown that NRF2 activation stimulates viral replication in cancer cells and disrupts the type I interferon response via increased autophagy^16^. In contrast to NRF2, the levels of which are primarily regulated by ubiquitination and proteasomal degradation, the protein turnover of KEAP1 is controlled by autophagy^17^. Heat shock, the classical activator of HSF1, induces mild activation of autophagy in cultured human A549 lung adenocarcinoma epithelial cells^18^. Notably, this occurs under conditions of persistent NRF2 activation due to loss-of-function mutations in KEAP1^19^. It has also been reported that HSF1 is required for autophagy induced by chemotherapeutic agents^20^. Upon accumulation of protein aggregates, HSF1 facilitates casein kinase 1-mediated phosphorylation of p62, whereas inhibition of HSF1 impairs protein aggregate-induced autophagosome formation^21^. HSF1 is often constitutively active in cancer cells and established human tumors^22^, and the prototypic HSF1-downstream target, inducible heat shock protein 70 (Hsp70), has been shown to exhibit tumor-specific localization to lysosomes and promote autophagy^23^. Furthermore, autophagy-inducing stress caused by histone deacetylase inhibition leads to an increase in the intracellular levels of acetylated Hsp70, which is required for the formation of the autophagosome^24^.

We have previously reported that the *ortho*-hydroxylated double Michael acceptor *bis*(2-hydoxybenzylidene)acetone (HBB2) is a potent dual inducer of the phase 2 response and the heat shock response^25^. In the present study, we found that HBB2 inhibits the development of cutaneous squamous cell carcinoma caused by chronic exposure to UV radiation in SKH-1 hairless mice. We further show that in addition to inducing the phase 2 response and the heat shock response, HBB2 is also an inducer of autophagy. This compound served as a chemical probe to elucidate mechanistically the role of transcription factors NRF2 and HSF1, the master regulators of these cytoprotective responses, in autophagy. We discovered that, in contrast to NRF2, which promotes autophagic flux, HSF1 suppresses autophagy. The effect of these two pathways on autophagy was closely mirrored by their opposing transcriptional regulation of p62. Understanding the regulation of autophagy in the context of enhanced HSF1 and NRF2 signaling is essential for the design of successful therapeutic strategies targeting this fundamental biological process.

## Results

### HBB2 inhibits tumor incidence and multiplicity in a mouse model of high-risk UV radiation-mediated cutaneous squamous cell carcinoma

**Figure 1:**
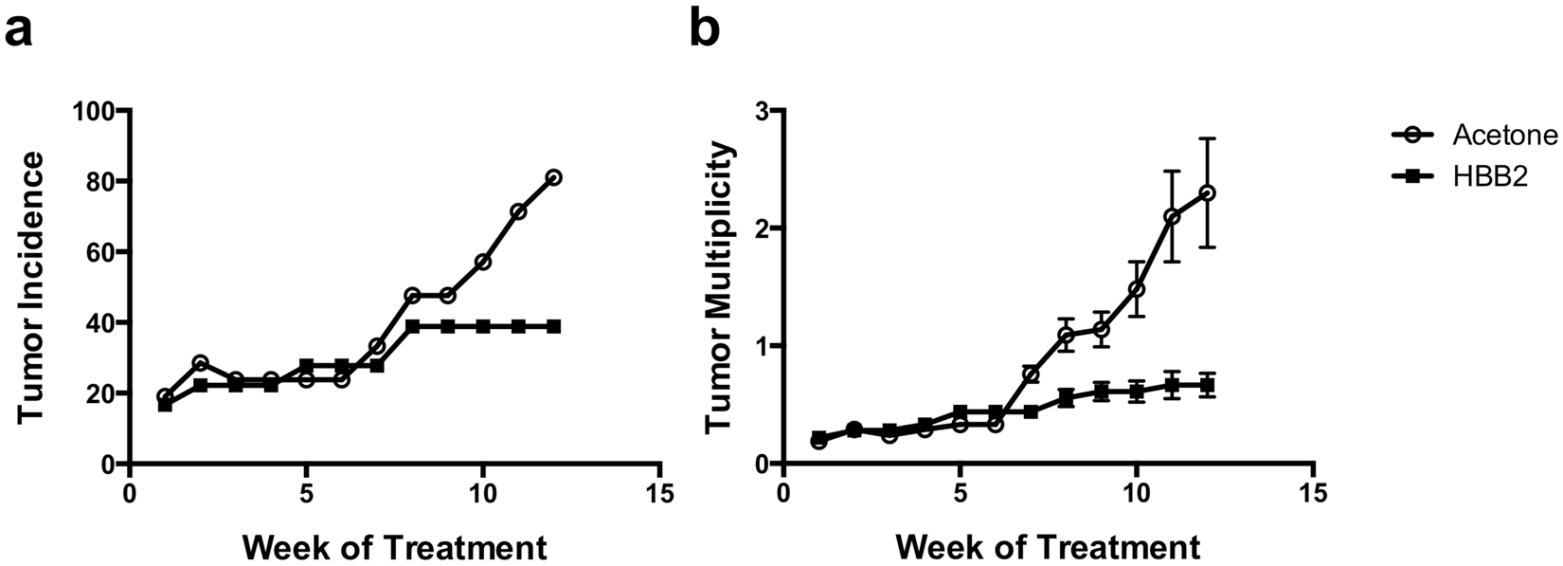
HBB2 inhibits tumor incidence and multiplicity in a mouse model of high-risk UV radiation-mediated cutaneous squamous cell carcinoma. Female SKH-1 hairless mice were exposed to UVB radiation (30 mJ/cm^2^/session) twice a week for 17 weeks, and then distributed into two groups. The mice from each group were treated topically with either 100 μl of 80% acetone (vehicle control group, n = 21) or 1 μmol of HBB2 dissolved in 100 μl of 80% acetone (HBB2-treatment group, n = 18) twice a week, for 12 weeks. Tumor incidence (**a**) is represented as the percentage of tumor-bearing animals in each group. Tumor multiplicity (**b**) is expressed as the total number of tumors in each group divided by the number of animals at risk; the data represent mean values ± SEM.

The role of activation of NRF2 and HSF1 during the process of carcinogenesis is context dependent, and both tumor prevention as well as tumor promotion effects have been described^26–28^. To determine the effect of HBB2, a potent dual inducer of NRF2 and HSF1^25^, on tumor development we used a mouse model of high-risk UV radiation-mediated cutaneous squamous cell carcinoma^29, 30^. Two groups of SKH-1 hairless mice were exposed chronically to low levels of UV radiation twice a week for 17 weeks. Irradiation was then discontinued. During the subsequent 12 weeks, one of the groups received topical applications of 1 μmol HBB2 twice a week, and the other group, vehicle (80% acetone) with the same frequency and duration. Tumor incidence and multiplicity were profoundly decreased in the HBB2-treated group. Thus, at the end of the experiment, 81% of the animals in the vehicle control group had developed at least one tumor, whereas this number was reduced to 40% in the HBB2-treatment group (Fig. 1a). The difference in tumor incidence between the two groups was statistically significant (*p* < 0.05, by ANOVA). Similarly, tumor multiplicity was reduced by ~70% (*p* < 0.05, by ANOVA), and at the end of the experiment, the number of tumors per mouse at risk was 2.3 and 0.66 for the vehicle- and the HBB2-treatment group, respectively (Fig. 1b). Overall, we conclude that in this mouse model of cutaneous squamous cell carcinoma, which is initiated by one of the most relevant human carcinogens and is characterized by an extraordinary high mutation burden^31, 32^, HBB2 inhibits the development of initiated tumors.

### HBB2 induces HSE-dependent transcription

**Figure 2:**
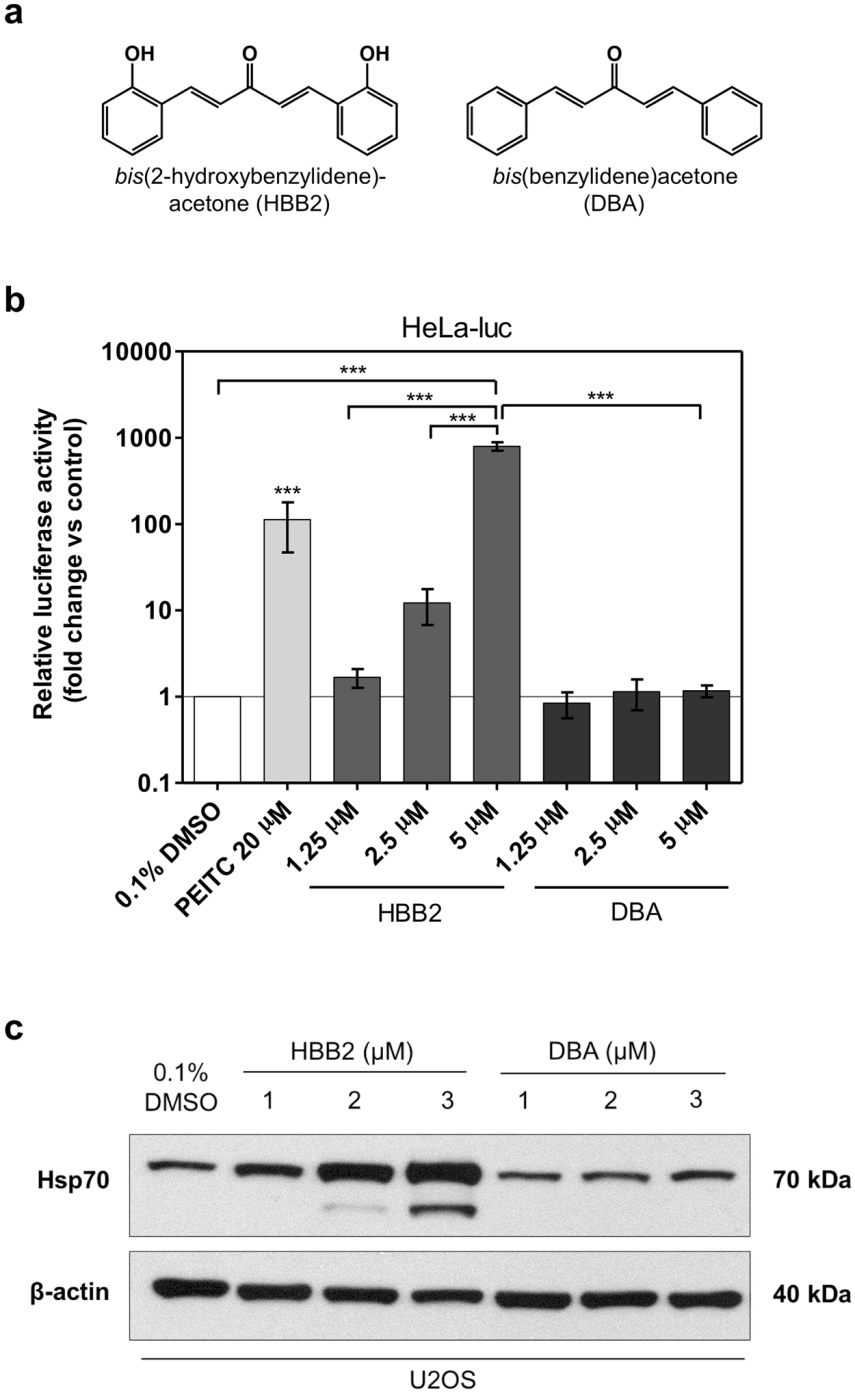
HBB2 robustly induces the heat shock response. (**a**) Chemical structures of HBB2 and DBA. (**b**) Luciferase gene reporter assay was performed in HeLa-luc cells treated with up to 5 µM of either HBB2 or DBA for 24 h. PEITC was included as a positive control. Luminescence values were normalised to respective vehicle controls (0.1% DMSO for HBB2 and DBA; 0.1% ACN (not included in the graph) for PEITC). Data are means ± SD from two independent experiments (n = 6 for each treatment group). ***p < 0.001 (one-way ANOVA and Tukey’s post-test). (**c**) Western blot analysis demonstrating a concentration-dependent increase in total Hsp70 levels in U2OS whole-cell lysates caused by a 24-h treatment with HBB2 but not with DBA. 0.1% DMSO was used as a vehicle control and β-actin acted as a loading control.

HBB2 reacts readily with sulfhydryl groups, whereas its non-hydroxylated structural analog *bis*(benzylidene)acetone (DBA) (Fig. 2a) has a much weaker reactivity^25, 33^. We tested the ability of HBB2 and DBA to activate HSF1-mediated transcription using the HeLa HSE-luciferase reporter cell line stably transfected with luciferase under the transcriptional control of the *HSP70.1* promoter containing heat shock elements, the upstream regulatory sequences to which HSF1 binds to initiate transcription^34^. At the highest concentration tested (5 µM), HBB2 produced a dramatic (~800-fold, *p* < 0.001) increase in luciferase activity (Fig. 2b). This effect is concentration-dependent, with 1.25 µM HBB2 causing a 1.7-fold increase, and 2.5 µM HBB2 increasing the reporter activity by 12-fold. In sharp contrast, no induction was observed in cells exposed to the structural analog DBA. Notably, the magnitude of the effect of HBB2 on HSE-mediated transcription exceeded that of the positive control, phenethyl isothiocyanate (PEITC)^35^.

Western blot analysis in the human osteosarcoma cell line U2OS confirmed the relatively steep concentration-response relationship for the ability of HBB2 to induce the endogenous Hsp70, with 1-, 2- and 3 µM HBB2 causing an increasingly greater upregulation in Hsp70 (Fig. 2c). Interestingly, a new, faster-migrating band was also observed upon treatment with increasing concentrations of HBB2. By use of in-gel tryptic digestion and mass spectrometry, we confirmed that this band was an isoform of Hsp70 (not shown). Together, these results demonstrate that HBB2 is a robust activator of the HSF1-mediated transcriptional responses.

### HBB2 increases the levels of p62 and LC3B

**Figure 3:**
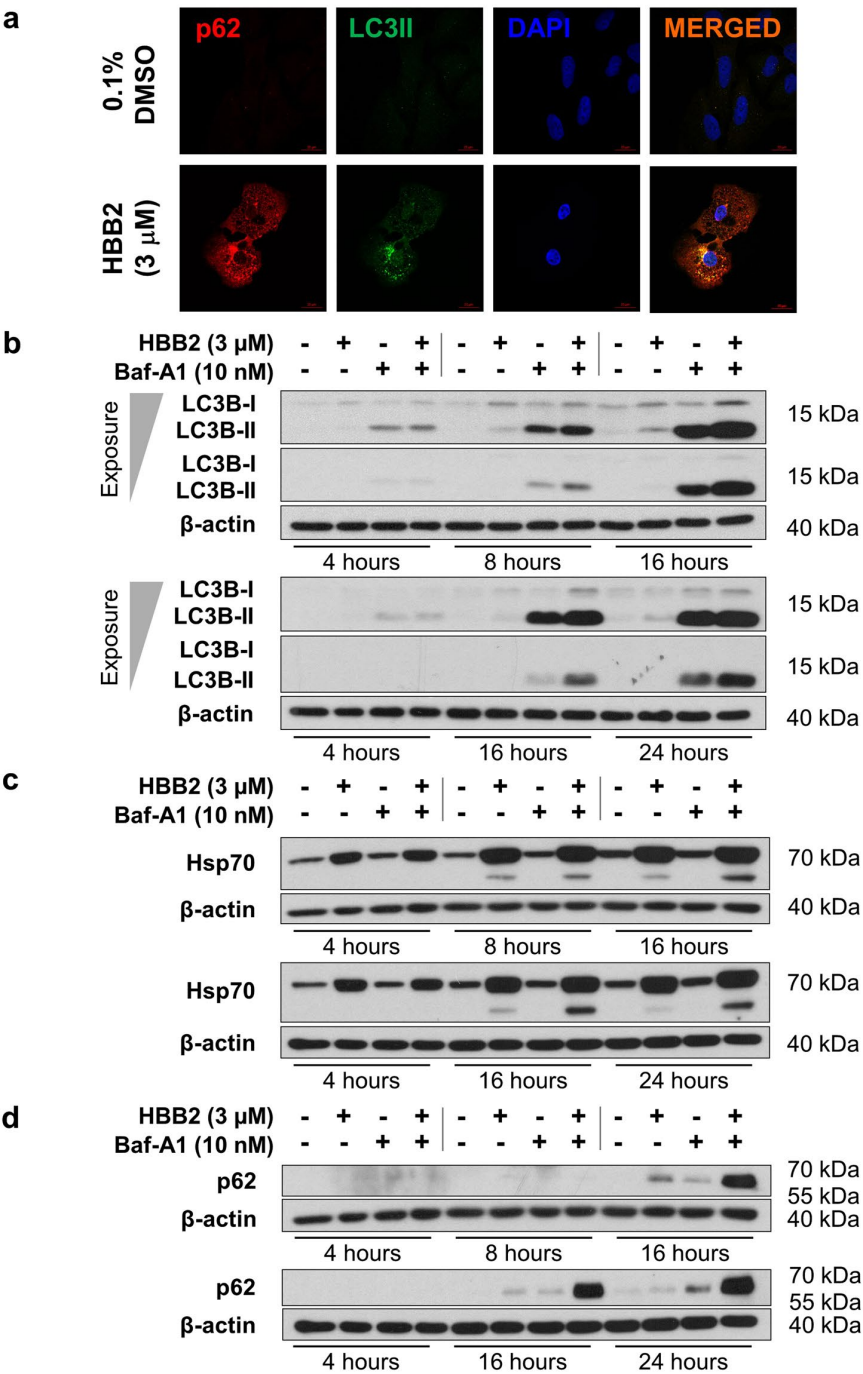
HBB2 causes lipidation of LC3B and accumulation of p62, and enhances autophagic flux. (**a**) Immunofluorescence analysis for p62 and LC3B in U2OS cells that had been exposed to HBB2 (3 µM) (bottom) or treated with vehicle (top) for 16 h. The images were obtained by confocal microscopy. Scale bar = 20 µm. (**b–d**) Western blot analysis of U2OS whole-cell lysates was conducted to detect the changes in the levels of LC3B-II (**b**), Hsp70 (**c**) and p62 (d) in response to a 4-, 8-, 16-, or 24 h treatments with HBB2 (3 µM) and/or bafilomycin A1 (Baf-A1; 10 nM), as indicated above the lanes. DMSO (0.1%, v/v) was used as a vehicle control (indicated as −/− above the blots), whereas β-actin levels served as a loading control.

Because of the individual links between the cellular stress responses regulated by HSF1 and NRF2 with autophagy, we next examined the potential ability of HBB2 to affect autophagy. We began by evaluating two biomolecular markers of autophagy – p62 and the microtubule-associated protein light chain 3 isoform B (LC3B). LC3B-II is a membrane-associated form of LC3B that arises from the processing of the soluble LC3B-I by conjugation with phosphatidylethanolamine; this lipidation process in turn allows association with both the inner and the outer surfaces of the autophagosome. Immunofluorescence analysis showed that exposure to HBB2 (3 μM) for 16 h caused an increase in the levels of p62 and LC3B, and further revealed that the two proteins co-localize in the cytoplasm of HBB2-treated cells (Fig. 3a).

These results strongly suggest that HBB2 affects the autophagic process. However, it was not clear whether autophagy was induced or inhibited. This is because autophagy is a dynamic process with the amount of autophagosomes (and their components, including LC3B-II and p62) present at any one time being dependent on their rate of formation as well as on their rate of degradation. To rule out a decrease in autophagosomal turnover as the reason for the increased levels of p62 and LC3B-II, experiments were carried out in the presence or absence of the autophagy inhibitor bafilomycin A1 (Baf-A1). When autophagy is induced, Baf-A1 causes accumulation of p62 and LC3B-II, whereas no further increase upon Baf-A1 addition is seen when autophagy is inhibited. Thus including Baf-A1 provides a measure of autophagic flux^36^. We therefore next evaluated the effect of HBB2 on autophagic flux in the presence or absence of Baf-A1.

### HBB2 increases autophagic flux

When HBB2 and/or Baf-A1 were applied to the cells for 4, 8, 16, or 24 h, a time-dependent increase in the levels of LC3B-II (Fig. 3b), Hsp70 (Fig. 3c), and p62 (Fig. 3d) was observed. In the case of LC3B-II, the upregulation by HBB2 was most pronounced at the 16-h time point (Fig. 3b). Moreover, when Baf-A1 was used in combination with HBB2, a cumulative effect of the two agents was seen, especially at the later time points (16 and 24 h). Since Baf-A1 is a specific inhibitor of the vacuolar-type H^+^-ATPase and prevents maturation of autophagic vacuoles by inhibiting the fusion between autophagosomes and lysosomes in the late phase of autophagy^37^, the cumulative effect on the increase in LC3B-II levels upon the combined treatment of the cells with HBB2 and Baf-A1 indicated an upregulation of autophagic flux by HBB2. This was further supported by changes in lysosomal distribution, from relatively uniform peripheral localization to predominantly asymmetric perinuclear clustering, as revealed by immunostaining for the lysosome-associated membrane protein 1 (LAMP1) (Fig. S1). Such lysosomal repositioning plays a critical role in the control of autophagic flux^38^.

Induction of Hsp70 by HBB2 was already clearly evident at the 4-h time point (Fig. 3c), with a gradual further increase throughout the time course of the experiment. Baf-A1 alone had no effect on the levels of Hsp70. However, in combination with HBB2, Baf-A1 caused a further increase in the intensity of the faster-migrating band, which was detected with the anti-Hsp70 antibody at the 16- and 24-h time points, and its accumulation upon inhibition of autophagy by Baf-A1 suggests that it may be involved/processed during autophagy. This is consistent with the previously reported localization of Hsp70 in lysosomes during autophagy^23^, and the requirement for this heat shock protein for the formation of the autophagosome^24^.

In contrast to Hsp70, the increase in p62 levels was only detectable at the 16-h time point (Fig. 3d), with a very substantial cumulative induction by the combined (HBB2 and Baf-A1) treatment. This enhanced accumulation of the autophagy-cargo protein upon the combined HBB2 and Baf-A1 treatment, compared to either treatment alone strengthens the conclusion that HBB2 is an inducer of autophagy. Such synergistic upregulation in the levels of p62 by the combination of HBB2 and Baf-A1 is most likely due to the fact that p62 is degraded by autophagy^39^, which is blocked by Baf-A1, whereas the increase in p62 levels caused by HBB2 alone could represent transcriptional upregulation of p62 caused by HBB2-dependent activation of NRF2^11^ or HSF1^12^, or both.

### NRF2 promotes, whereas HSF1 inhibits autophagic flux

**Figure 4:**
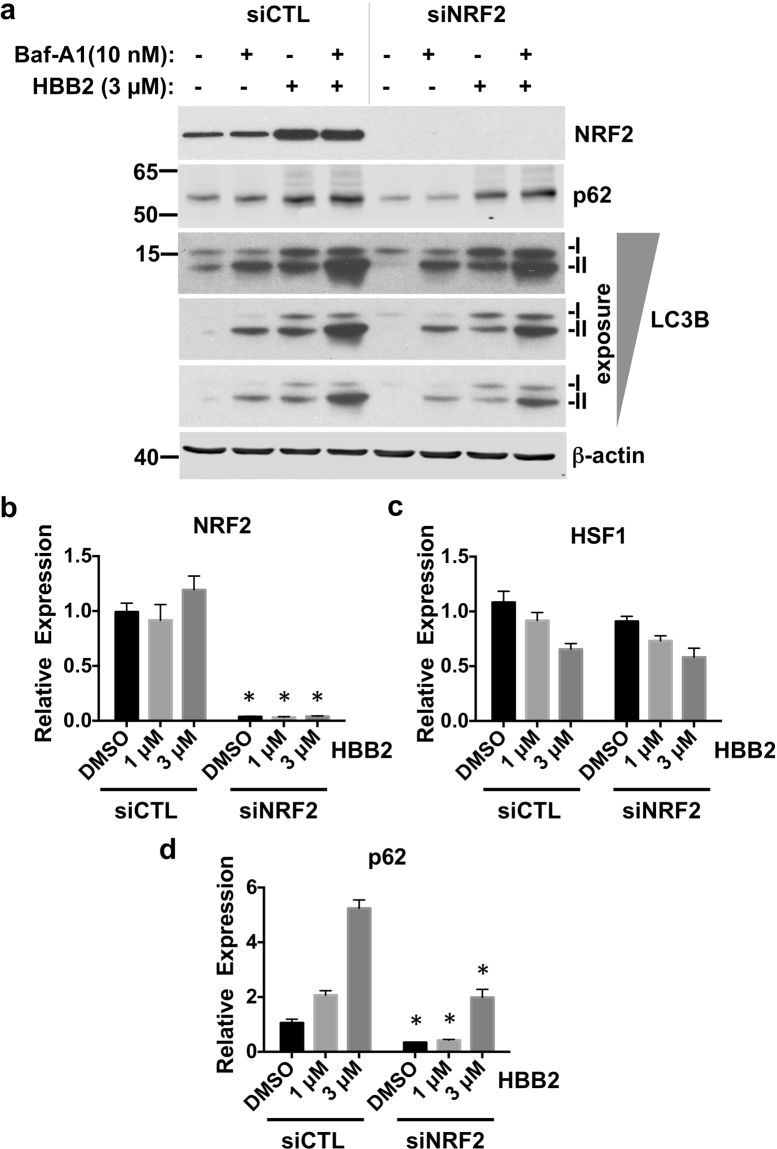
Knockdown of NRF2 inhibits autophagy. (**a**) Immunoblotting analysis of NRF2, p62 and LC3B in lysates from U2OS cells, which had been transfected with either control siRNA (siCTL) or NRF2 siRNA (siNRF2) for 48 h, and subsequently treated with vehicle (0.1% DMSO) or HBB2 (3 µM) for 18 h, and supplemented with 10 nM bafilomycin A1 (Baf-A1) or vehicle (0.1% DMSO) for the last 2 h. The levels of β-actin served as a loading control. For detection of LC3B, proteins from cell lysates were resolved using 13% SDS-PAGE and transferred onto 0.45 µm PVDF membranes, whereas for detection of NRF2 and actin, proteins were blotted onto 0.45 µm NC membranes. (**b–d**) Real-time PCR analysis of NRF2 (**b**), HSF1 (**c**), and p62 (**d**) from a parallel experiment described in (**a**) except without Baf-A1 treatment. The mRNA levels of 18 S were used for normalization. The data are represented by mean + SD from three independent transfections. Student’s t-test was used to test for statistical significance, where *represents *p* < 0.05 when comparing siCTL and siNRF2 for each treatment pair.

**Figure 5:**
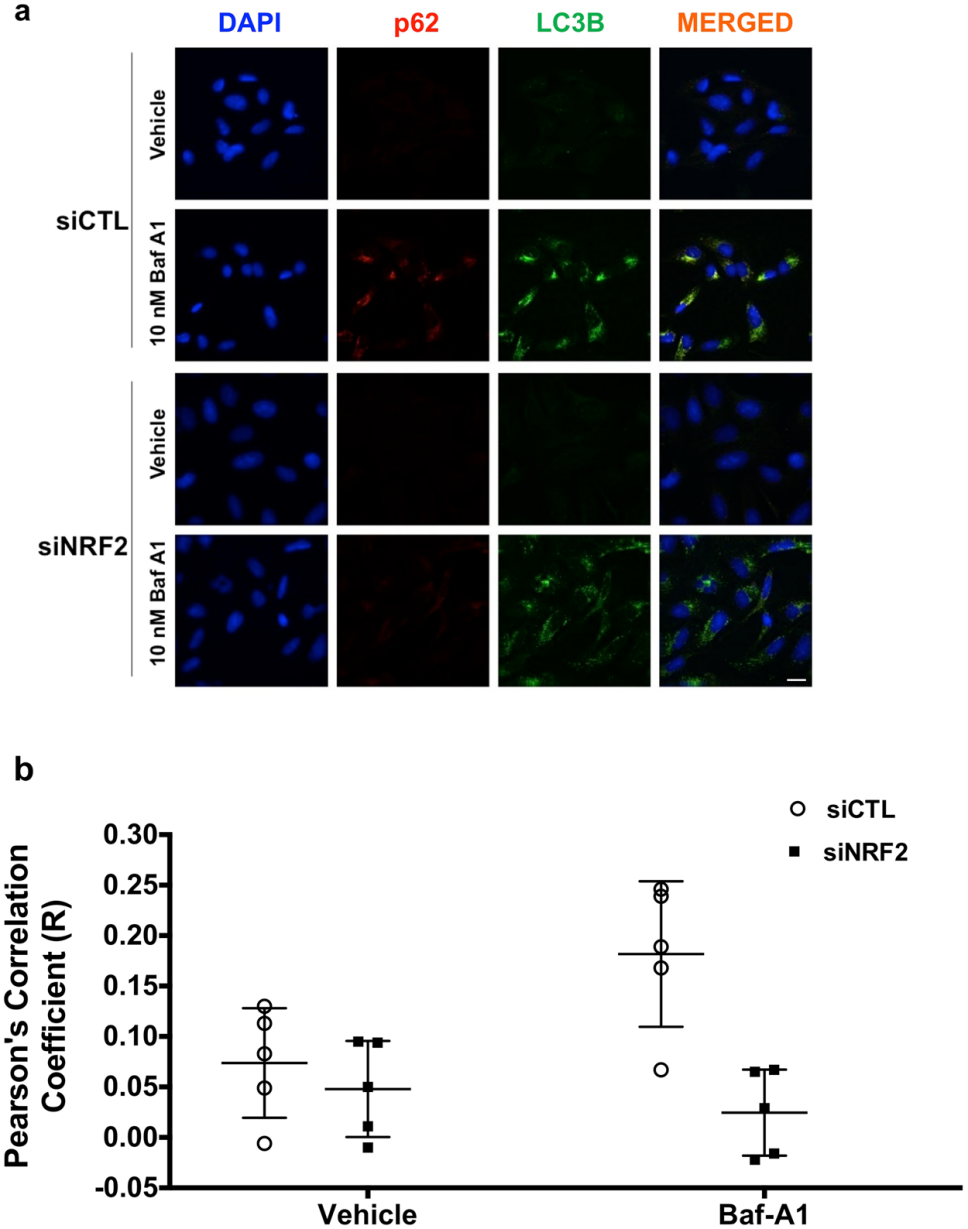
NRF2 is required for basal and HBB2-induced autophagic flux. Immunofluorescence images (**a**) and quantitative analysis (**b**) of the colocalization of p62 and LC3B in U2OS cells, which had been transfected with either control siRNA (top two panels) or NRF2 siRNA (bottom two panels) for 48 h, and subsequently treated with bafilomycin A1 (Baf-A1; 10 nM) for 18 h. Note that in the case of detection of LC3B, an antibody exhibiting a stronger reactivity to the lipidated form (LC3B-II) was used (LC3B D11, CST #3868). Wide-field microscope (Deltavision Elite) was used to collect the images where in each field 25 images were taken with an optical section of 0.2 μm. The deconvolved images represent the summed intensity projection using SoftWorX (Version 5.5). Scale bar = 20 µm.

Having established that, in addition to activating NRF2 and HSF1, HBB2 also induces autophagy, we next assessed the contribution of NRF2 and HSF1 to the HBB2-mediated induction of autophagy. To this end, we depleted each transcription factor individually using siRNA. Immunoblotting (Fig. 4a) and quantitative real-time PCR (Fig. 4b) analyses showed that the levels of NRF2 were reduced by >90% in cells transfected with the NRF2 siRNA. Interestingly, for reasons that are currently unknown, the levels of HSF1 mRNA were reduced by the treatment with HBB2, although there was no significant difference between control siRNA (siCTL)- and siNRF2-transfected cells (Fig. 4c). In close agreement with the results in untransfected cells (Fig. 3a,b), the levels of LC3B-II were increased after treatment with HBB2 in cells transfected with siCTL (Fig. 4a). Notably, the level of LC3B-II in HBB2-treated cells was much lower when NRF2 was knocked-down (siNRF2, Fig. 4a), indicating that NRF2 mediates, at least in part, the activation of autophagy by HBB2. Immunoblotting analysis also revealed that depletion of NRF2 notably reduced both basal and HBB2-induced levels of p62 protein (Fig. 4a). Immunofluorescence imaging of U2OS cells that were transfected with either control or NRF2 siRNA, and subsequently treated with vehicle or Baf-A1 confirmed the requirement for NRF2 for basal autophagic flux (Fig. 5a). Quantitative analysis of the image data further revealed that the co-localization of p62 and LC3B was significantly reduced under conditions of NRF2 deficiency, and this was especially pronounced when autophagy was blocked with Baf-A1 (Fig. 5b).

We further asked whether NRF2 directly activates transcription of p62 in response to HBB2. Upon exposure to HBB2, the levels of NRF2 were increased (Fig. S2a), in agreement with the known NRF2 inducer activity of HBB2. The mRNA levels of the NRF2-target genes heme oxygenase 1 (HMOX1) (Fig. S2b), aldo-keto reductase 1C1 (AKR1C1) (Fig. S2c), and aldo-keto reductase 1B10 (AKR1B10) (Fig. S2d) were correspondingly and dose-dependently upregulated. In cells transfected with non-targeting siRNA (siCTL), HBB2 treatment induced a dose-dependent increase in p62 mRNA (Fig. 4d). Importantly, compared to cells transfected with the control siRNA (siCTL), the basal and the inducible mRNA levels of p62 were diminished in cells transfected with the NRF2 siRNA (Fig. 4d).

Together, these results imply that NRF2 is a mediator of the activation of autophagy by HBB2.

**Figure 6:**
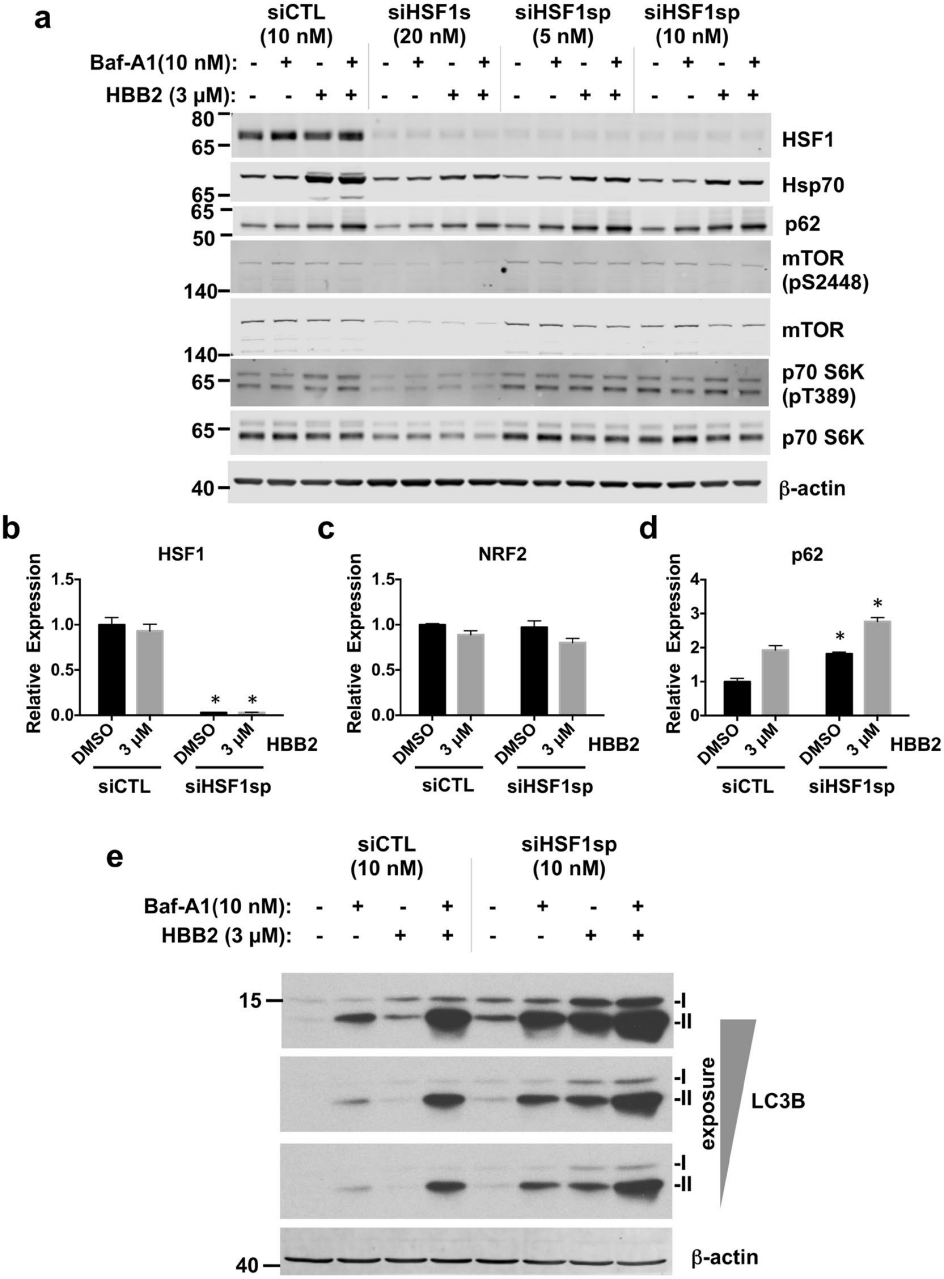
HSF1 inhibits autophagic flux. (**a**) Immunoblotting analysis of HSF1, Hsp70, p62, mTOR, phosphorylated mTOR (pS2448), p70 S6K, phosphorylated p70 S6K (pT389) in lysates from U2OS cells, which had been transfected with either control siRNA (siCTL) or HSF1 siRNA (siHSF1s or siHSF1sp) for 48 h, and subsequently treated with vehicle (0.1% DMSO) or HBB2 (3 µM) for 18 h, and supplemented with bafilomycin A1 (Baf-A1; 10 nM) or vehicle (0.1% DMSO) for a the last two hours of HBB2 treatment. The levels of *β*-actin served as a loading control. (**b–d**) Real-time PCR analysis of HSF1 (**b**), NRF2 (**c**), and p62 (**d**) from a parallel experiment described in (**a**) except without Baf-A1 treatment, in U2OS cells transfected with siCTL (10 nM) or siHSF1sp (10 nM). The mRNA levels of 18 S were used for normalization. The data are represented by means + SD from three independent transfections. Student’s t-test was used to test for statistical significance, where *represents *p* < 0.05 when comparing siCTL and siHSF1sp for each treatment pair. (**e**) Immunoblotting analysis of LC3B in lysates from U2OS cells, which had been transfected with either siCTL (10 nM) or siHSF1sp siRNA (10 nM) for 48 h, and subsequently treated with vehicle (0.1% DMSO) or HBB2 (3 µM) for 18 h, supplemented with bafilomycin A1 (Baf-A1; 10 nM) for the last 2 h. The levels of β-actin served as a loading control.

We then examined the contribution of HSF1 to the HBB2-mediated activation of autophagy using cells transfected with HSF1 siRNA. We depleted HSF1 using either an individual HSF1-targeting siRNA^40^, designated here as siHSF1s, or an siRNA SMARTpool of four individual siRNAs (Dharmacon) (siHSF1sp). In both cases, the depletion resulted in more than 90% reduction in HSF1 at the protein (Fig. 6a) and the mRNA (Fig. 6b) levels. As expected, the mRNA levels of NRF2 were not affected (Fig. 6c). We further determined how loss of HSF1 affects markers of autophagy, such as lipidation of LC3B and p62 in response to HBB2, in the absence or the presence of Baf-A1- mediated block of their degradation. Both approaches (Figs 6e and S3) showed that HSF1 depletion dramatically increased basal autophagic flux. Furthermore, siHSF1sp-mediated depletion of HSF1 also strongly promoted autophagy induced by HBB2. Also specific for siHSF1sp was an increase in p62 protein level in cells treated with HBB2 (Fig. 6a).

We next considered the mechanism by which siHSF1 causes upregulation of autophagy. mTOR is a key regulator of autophagy, and its inhibition is known to mediate autophagic response to nutrient/amino acid deprivation^41^. In yeast, HSF1 activation inhibits mTOR signalling^42^. To determine whether the mTOR status was altered by HSF1 depletion in U2OS cells, we assessed mTOR phosphorylation at S2448, a marker for active mTOR, and its downstream effector, p70 S6K (phosphorylated at T389) in U2OS cells treated with HSF1-targeted siRNAs (Fig. 6a). No changes in the mTOR pathway were detected in cells treated with siRNA SMARTpool directed against HSF1, suggesting that HSF1 depletion promotes autophagy independently on mTOR. Surprisingly, the total protein level of both mTOR and p70S6K, as well as of their phosphorylated forms, were reduced in U2OS cells that were treated with siHSF1s siRNA. This was not mimicked by the reduction in the levels of Hsp90α and/or Hsp90β using siRNA that recapitulates some of the downstream effects of HSF1 depletion (Fig. S4). We hypothesized that an off-target reduction in protein translation by siHSF1s siRNA might be responsible for such changes, and therefore further focused on the results of HSF1 depletion obtained with the siRNA SMARTpool against HSF1.

Another important mediator of both mTOR-dependent and mTOR-independent autophagy is p62^43^. To assess whether HSF1 could modulate HBB2-induced autophagy via transcriptional regulation of p62, we measured the mRNA levels of p62 in HSF1-depleted U2OS cells treated with HBB2 or DMSO vehicle (Fig. 6d). Surprisingly, the knockdown of HSF1 caused an increase (by ~2-fold) in the transcription of p62 under both basal as well as HBB2-induced conditions (Fig. 6d), consistent with the enhanced autophagy observed in HSF1-depleted cells.

Together, these results demonstrate that NRF2 promotes, whereas HSF1 inhibits autophagy, and identify NRF2 activation as the principal mediator of the activation of autophagy by HBB2.

## Discussion

Our results suggest that autophagy is induced by HBB2 predominantly via NRF2-dependent transcriptional upregulation of p62, despite a strong co-activation of HSF1 that is inhibitory for p62 expression. How could this apparent paradox be explained? It is possible that while p62 transcription is inhibited by the presence of HSF1, the activation of this pathway by the drug may not result in further inhibition of p62, thus shifting the balance between NRF2-mediated upregulation and HSF1-mediated inhibition of p62 towards the former. This is supported by the fact that HBB2 does not cause a decrease in p62 expression in cells with >90% depletion of NRF2 (Fig. 4d). The induction of p62 by HBB2 in these cells must be due to the residual NRF2 activity. An alternative explanation could be that HSF1 activation by HBB2 switches HSF1 from an inhibitor to an activator of p62; however, the ability of HSF1 depletion to increase in p62 mRNA level in HBB2 treated cells (Fig. 6d) argues against it - rather, HSF1 appears to act as a “brake” to p62 expression (and, consequently, to autophagy), regardless of the presence of HBB2.

In conclusion, the *ortho*-hydroxylated double Michael acceptor *bis*(2-hydoxybenzylidene)acetone (HBB2), a potent dual inducer of the phase 2 response and the heat shock response, is also an inducer of autophagy. This finding provided a chemical probe for investigating mechanistically how autophagy is activated in the context of enhanced HSF1- and NRF2-mediated signaling. Collectively, our results show that HBB2 induces autophagy by activation of NRF2. In contrast to NRF2, which promotes autophagic flux, HSF1 suppresses it. Although it is not clear at present whether the observed inhibition of the development of cutaneous tumors in the animal experiment is mediated by NRF2 and/or HSF1, or induction of autophagy, this experiment demonstrates that activation of the cellular stress responses is an effective strategy for protection against UV radiation-mediated skin carcinogenesis.

## Methods

### Compounds

Phenethyl isothiocyanate (PEITC), *bis*(benzylidene)acetone (DBA) and bafilomycin A1 (Baf-A1) were from Sigma Aldrich. *Bis*(2-hydroxybenzylidene)acetone (HBB2) was synthesized by Dr. Ronald Hicks (California State University East Bay, USA). PEITC was dissolved in acetonitrile (ACN), whereas HBB2, DBA and Baf-A1 were dissolved in dimethyl sulfoxide (DMSO). Prior to use, the compounds were diluted (1:1000) in the cell culture medium to achieve the appropriate final concentrations (indicated in the text). The final concentration of the solvent in the cell culture medium was maintained at 0.1% (v/v).

### Antibodies

Solutions of primary antibodies were prepared in 5% non-fat milk in PBST, unless indicated otherwise. The following antibodies were used: mouse monoclonal anti-β-actin, 1:10000–1:20000, Sigma; mouse monoclonal anti-Hsp70, (clone C92F3A-5) 1:1000, StressMarq; mouse monoclonal anti-Hsp90α/β, (H90–10) 1:5000, Alexis Biochemicals; mouse anti-Hsp90, BD Transduction Laboratories, 1:5000; rabbit monoclonal anti-LC3B, 1:1000, Sigma; rabbit monoclonal anti-LC3B (D11), 1:1000, Cell Signaling Technology (CST); mouse monoclonal anti-SQSTM1/p62, 1:5000, Abcam; rabbit polyclonal anti-HSF1, 1:1000, Enzo Life Sciences; rabbit monoclonal anti-mTOR (7C10), 1:1000, CST; rabbit monoclonal anti-mTOR (pS2448), CST; rabbit monoclonal anti-p70 S6K, CST; rabbit monoclonal anti-p70 S6K (pT389), CST; rabbit polyclonal anti-4E-BP1, 1:1000, CST (#9452S), rabbit polyclonal anti-p4E-BP1 (pT36/47), 1:1000 in 5% BSA in PBST, CST (#9459); rabbit polyclonal anti-LAMP1, 1:200, Caltag Medsystems (PSI-3629); and rabbit monoclonal anti-NRF2 (D1Z9C), CST.

### Cell culture

Human osteosarcoma U2OS cells, a kind gift from Sonia Rocha (School of Life Sciences, University of Dundee, UK), were cultured in Dulbecco’s Modified Eagle Medium (DMEM; Gibco, Life Technologies) that contains L-glutamine, sodium pyruvate, and high D-glucose content (4.5 g/L), supplemented with 10% (v/v) heat inactivated fetal bovine serum (FBS; Thermo Scientific). The stable HeLa-luc reporter cell line that expresses luciferase under the control of the HSP70.1 promoter, developed as described in ref. ^44^, was a kind gift from Richard Morimoto (Northwestern University, USA) and was cultured in the same DMEM basal medium supplemented with 10% (v/v) heat-inactivated FBS and 600 µg/ml G418 (both from Gibco, Life Technologies). All cell lines were maintained in a humidified incubator (Heracell 150, Thermo Scientific) at 37 °C and 5% CO_2_. Unless stated otherwise, the cells were seeded into 6-well plates at a density of 2 × 10^5^ per well the day before treatment with the specified compounds.

### Whole-cell lysis and sample preparation for immunoblotting

**C**ells were lysed in non-reducing sample buffer (50 mM Tris-HCl pH 6.8, 2% (w/v) sodium dodecyl sulfate (SDS), 10% (v/v) Glycerol, and 0.02% (w/v) Bromophenol blue) using 150 µl of the buffer for each well of a standard 6-well plate (Nunclon, Thermo Scientific). Whole-cell lysates were then collected in fresh Eppendorf tubes, optionally boiled at 100 °C for 5–10 minutes, and sonicated using Vibra-Cell ultrasonic processor (Sonic), prior supplementing the lysates with DTT up to 0.1 M final concentration. The samples were snap frozen and stored at −20 °C until analysis. Proteins in the lysates were resolved by SDS-polyacrylamide gel electrophoresis (PAGE) and then transferred onto either nitrocellulose (NC) or polyvinylfluoridine (PVDF) membranes. The membranes were blocked in 5% (w/v) non-fat milk (Marvel) dissolved in PBS containing 0.1% (v/v) Tween 20 (PBST) for 1 h, and subsequently incubated with the respective primary antibody solutions either at room temperature for 2 h or at 4 °C overnight, with continuous agitation. Following incubation with primary antibodies, the NC or PVDF membranes were washed three times with PBST for at least 30 min to 1 hour, and subsequently incubated with the corresponding secondary antibodies (horseradish peroxidase (HRP)-conjugated goat anti-rabbit (GAR) antibody, 1:5000, (Bio-Rad/CST), or HRP-conjugated goat anti-mouse (GAM) antibody, 1:5000, Bio-Rad, or IRDye fluorescent dyes conjugated GAM/GAR-680RD or GAM/GAR-800CW, LI-COR). All blots shown are representative of at least three independent experiments.

### Luciferase reporter assay

HeLa-luc cells were seeded (1 × 10^4^ cells/well) into white flat-bottomed 96-well plates (Thermo Scientific). The following day, the cells were treated in 8 replicates with HBB2 or DBA (1.25, 2.5 or 5 µM), respective vehicles [0.1% (v/v) acetonitrile or 0.1% (v/v) DMSO], or 20 µM PEITC, which was used as a positive control. After 24 h, 50 µl of Bright-Glo luciferase assay reagent (Promega) was added directly into the medium in each well, followed by gentle shaking of the plates at room temperature for 5 minutes. The luminescence was then measured (1-sec read per well) in half of the wells treated with each compound using Modulus II microplate reader (Turner Biosystems). The media was removed from the other half of the plates. The cells were then washed twice with PBS, followed by addition of 50 µl of the lysis buffer [50 mM Tris-HCl pH 7.5, 10 mM KCl, 5 mM MgCl_2_, 0.5% Nonidet P40 (Roche) and 1 mM dithiothreitol (DTT)] into each well. Twenty microliters of the resulting lysate were used to determine its protein concentration and normalize the luminescence values.

### Generation of HSF1- and NRF2-knockdown U2OS cells

The expression of HSF1 and NRF2 was reduced by RNA interference. U2OS cells (5 × 10^5^) were reverse transfected and plated in 6-cm dishes with either Negative Control siRNA (siCTL) (AAUUCUCCGAACGUGUCACGU), human HSF1 single siRNA (CAGGUUGUUCAUAGUCAGAAU siRNA), human NRF2 siRNA (SMARTpool, ON-TARGETplus, #L-003755-00-0005, Dharmacon) or human HSF1 siRNA (SMARTpool siGENOME, #M-012109-01-0005, Dharmacon) using Lipofectamine RNAiMAX (Thermo) according to the manufacturer’s protocol. The single HSF1 siRNAs and the Negative Control siRNA were synthesized by Eurofins Genomics, Ebersberg Germany. On the following day, the transfected siCTL, siNRF2 and siHSF1 cells were replated in 6-well plates at a density of 3.5 × 10^5^, 4.5 × 10^5^ and 5.5 × 10^5^ cells per well, respectively, in complete medium, and incubated for a further 24 h. Forty eight hours after transfection, the cells were treated with DMSO (0.1%, v/v) or HBB2 for 16–18 h, and with Baf-A1 during last two hours of HBB2 treatment.

### Immunofluorescence

U2OS cells (2.5 × 10^5^ cells/well) were seeded on 18 × 18 mm glass coverslips (VWR) with a 1.5-mm thickness. Twenty hours later, cells were treated with either vehicle (0.1% DMSO) or 3 μM HBB2 for a further 16 h. Cells were then washed three times with PBS and fixed in ice-cold (−20°C) 100% methanol, and kept in the freezer for 15 min. The cells were washed three times with PBS and subsequently washed with 0.3% (v/v) Triton X-100 in PBS (PBS-Tx) for a further 15 min. Next, the cells were placed in blocking solution (3% normal donkey serum in PBS-Tx) for 30 min at room temperature (RT) after which they were incubated for 1 h at RT with rabbit anti-LC3B (1:200) (CST) and mouse anti-p62 (1:200) (Abcam) antibodies diluted in the blocking solution. The cells were subjected to three washes every 5 min using PBS-Tx, and then incubated with either FITC or Alexa Fluor 594  conjugated donkey anti-rabbit (1:400) and Alexa Fluor 594  or Alexa Fluor 680  conjugated donkey anti-mouse (1:400) secondary antibodies (Jackson Immunoresearch) diluted in the blocking solution for 1 h. After washing three times every 5 min using PBS-Tx and another wash for a further 5 min with PBS, the cells were incubated with DAPI (dissolved in PBS at a final concentration of 2.5 μg/ml) for 5 min at room temperature. The cells were quickly washed three times with PBS and were subsequently mounted onto glass slides (VWR) using 30 μl of ProLong gold antifade mounting medium (Invitrogen), and let to cure overnight at RT. On the following day, the samples were imaged using either a confocal microscope (LSM 710, Zeiss) or a wide-field microscope (Deltavision Elite, GE Healthcare). The imaging experiments were performed at least three times.

### Colocalization Analysis

Images were obtained using a Deltavision Elite wide-field microscope (GE Healthcare) with an Olympus 60x/1.42 Plan Apo lens and Coolsnap HQ camera. In each field, 25 optical sections were obtained with 0.2-µm thickness per section. The images were deconvolved using SoftWorX (version 5.5). The colocalisation analysis of LC3B and p62 was performed in Volocity (Perkin Elmer, version 6.3) using its colocalisation module. For each image, individual threshold values were determined for each of the channels (LC3B and p62) using 4 standard deviations above the mean intensity. The thresholded Pearson’s Correlation Coefficient (PCC) was used as a statistical parameter to measure colocalization. For each condition, images from 5 fields of view were obtained, where in each field there were at least 4 cells. This experiment was conducted twice.

### Skin carcinogenesis

The skin carcinogenesis experiment was in compliance with the National Institutes of Health Guidelines, and was approved by the Johns Hopkins University Animal Care and Use Committee. For this experiment, female SKH-1 hairless mice (4 weeks old) from Charles River (Wilmington, MA) were acclimatized for 2 weeks in a 12-h light/12-h dark cycle, 35% humidity, and free access to water and pelleted AIN 76 A diet (Harlan TekLad). UV radiation (a mixture of UVB and UVA wavelengths, 65% and 35% of the total energy, respectively) was provided by UV lamps (FS72T12-UVB-HO, National Biological Corporation, Twinsburg, OH). Two groups of 21 animals were exposed to UV radiation (30 mJ/cm^2^/session) twice weekly, from 7 to 24 weeks of age, during which time 3 mice died for reasons that were unrelated to the irradiation protocol. After completion of the irradiation schedule, the animals were treated topically on their dorsal skin twice a week for 12 weeks. The mice from one of the groups (n = 21) received 100 μl of 80% acetone (v/v), the animals from the second group (n = 18) received 1 μmol of HBB2 dissolved in 100 μl of 80% acetone (v/v). Tumors (defined as lesions >1 mm in diameter) and body weights were recorded weekly. Tumor volumes were determined by measuring the height, length, and width of each lesion. The average of the three measurements was used as the diameter, and the volume was calculated (v = 4πr^3^/3).

### Data analysis

All quantitative data are represented graphically and in the text as mean values ± 1 standard deviation (SD), except for tumor incidence and multiplicity values that are represented as mean values ± 1 standard error of means (SEM). One-way ANOVA and Tukey’s Multiple Comparison post-test or two-way ANOVA and Bonferroni post-test were used when analyzing the data.

## Electronic supplementary material

Supplementary Information
