# Supplementary material for: Transcription factors NRF2 and HSF1 have opposing functions in autophagy
Source: Sci Rep. 2017 Sep 8;7:11023. doi: 10.1038/s41598-017-11262-5 (PMC5591275; doi:10.1038/s41598-017-11262-5)
Supplement: Supplementary file 1 — Supplementary Information [file 41598_2017_11262_MOESM1_ESM.pdf]

Supplementary Information

Transcription factors NRF2 and HSF1 have opposing functions in autophagy

Sharadha Dayalan Naidu, Dina Dikovskaya, Egle Gaurilcikaite, Elena V. Knatko, Zachary R. Healy, Hema Mohan, Glenn Koh, Axel Laurell, Graeme Ball, David Olagnier, Laureano de la Vega, Ian G. Ganley, Paul Talalay, and Albena T. Dinkova-Kostova

Supplementary Materials and Methods

Quantitative real-time PCR. The primers and probes (TaqMan® Gene Expression Assays) used to measure the mRNA levels for HMOX1 (Hs01110250_m1), AKR1C1 (Hs01397413_m1), and AKR1B10 (Hs00252524_m1), SQSTM1/p62 (Hs01061917_g1), MTOR (Hs00234508_m1), NFE2L2 (Hs00975961_g1) and HSF1 (Hs01027616_g1) were from Life Technologies. Total RNA was extracted from U2OS cells using the RNeasy Kit (Qiagen Ltd.) according to the manufacturer's instructions. Omniscript RT Kit (Qiagen Ltd.) was then used to reverse-transcribe 500 ng of total RNA into cDNA. Real-time PCR was carried out on Perkin Elmer/Applied Biosystems Prism Model 7500 or the 7700 Sequence Detector instrument. The TaqMan data for the mRNA species were normalized using ACTB (β-actin) (Hs01060665-g1) or 18S (Hs03003631-g1) as an internal control.

Generation of Hsp90α and/or Hsp90β-knockdown U2OS cells. The expression of Hsp90 was reduced by RNA interference. U2OS cells were reverse transfected in 6-well plates (3×10^5 cells per well) with either Negative Control siRNA (siCTL) (AAUUCUCCGAACGUGUCACGU, Eurofins Genomics), or siRNA against human Hsp90α and/or Hsp90β (HSP90AA1 and/or HSP90AB1 genes, ON-TARGET plus SMARTpool, Dharmacon) using Lipofectamine RNAiMAX (Thermo) according to the manufacturer's protocol. Forty eight hours after transfection, cells were washed in PBS at room temperature, lysed in SDS sample buffer (62.5 mM Tris-HCl pH 6.8, 2%

(w/v) sodium dodecyl sulfate (SDS), 10% (v/v) Glycerol, 10 mM DTT and 0.02% (w/v) Bromophenol blue), and sonicated using Vibra-Cell ultrasonic processor (Sonic) for 3 pulses of 5 seconds at 20% amplitude. Proteins were resolved by SDS-PAGE on 4-12% polyacrylamide gels (Invitrogen) under MOPS buffer, alongside the PageRuler pre-stained protein ladder (Thermo Scientific), and transferred onto nitrocellulose membrane for immunoblot analysis as above.

Supplementary Figures

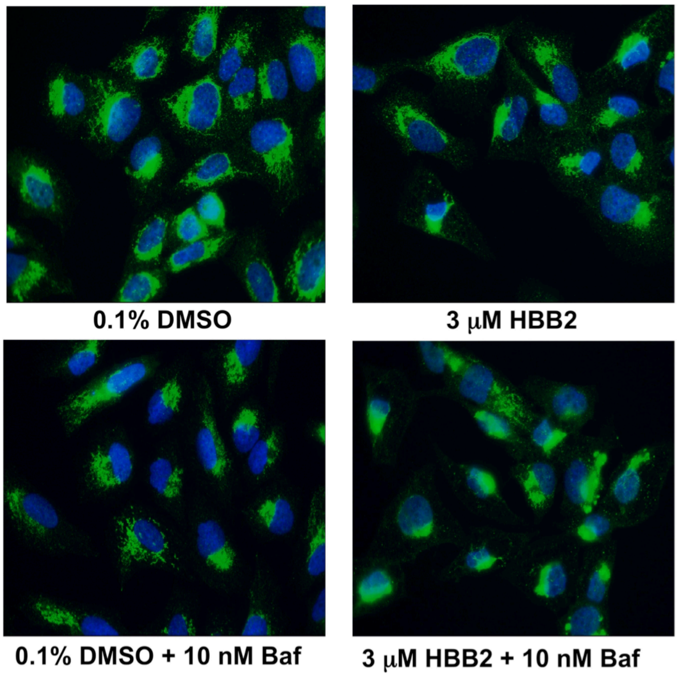

Figure S1. HBB2 causes redistribution of lysosomes. Immunofluorescence images of the lysosome-associated membrane protein 1 (LAMP1) in response to a 16-h treatment with HBB2 (3 μM) and/or bafilomycin A1 (Baf-A1; 10 nM).

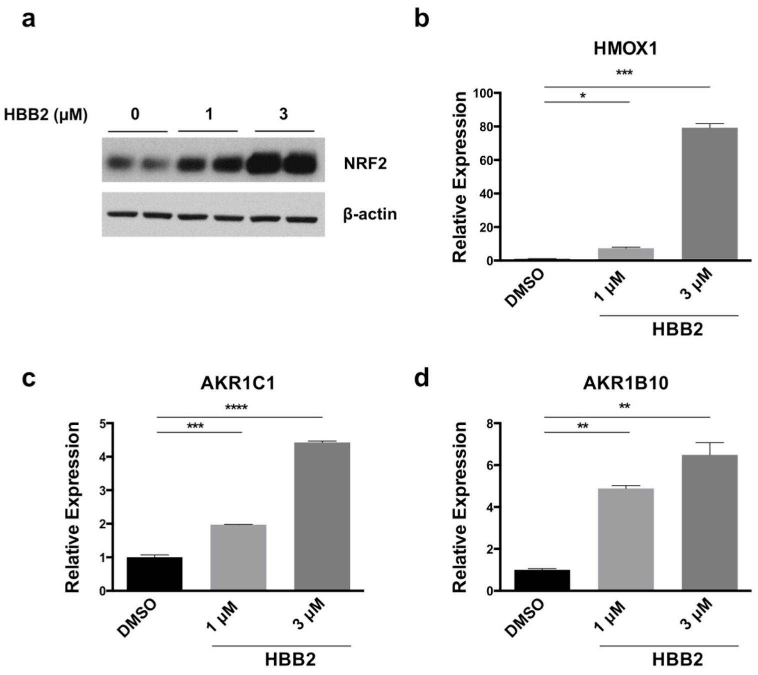

Figure S2. HBB2 activates NRF2. U2OS cells grown on 6-well plates were treated with HBB2 (1 or 3 μM). The protein levels of NRF2 **(a)** were evaluated by immunoblotting at 16 h post-treatment, and the levels of mRNA for HMOX1 **(b)**, AKRIC1 **(c)**, and AKR1B10 **(d)** were determined by quantitative real-time PCR at 24 h post-treatment. DMSO (0.1%, v/v) was used as a vehicle control. The levels of β-actin served as a loading control. *, $p < 0.05$, **, $p < 0.01$, ***, $p < 0.001$, ****, $p < 0.0001$ (n = 3, one-way ANOVA).

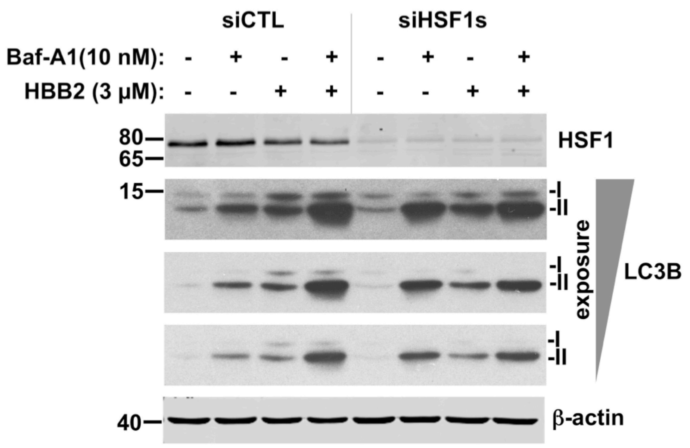

Figure S3. HSF1 inhibits autophagic flux. U2OS cells transfected for 48 h with 40 nM of non-targeting siRNA (siCTN) or an individual HSF1-targeting (siHSF1s) siRNA were treated for 18 h with 3 μM HBB2 or vehicle (0.1% DMSO), and supplemented with Bafilomycin A1 (Baf-A1, 10 nM) or vehicle (0.1% DMSO) for the last 2 h of treatment. Proteins from total cell lysates were separated by SDS PAGE and immunoblotted for LC3B (I and II) and HSF1. β-actin was used as a loading control.

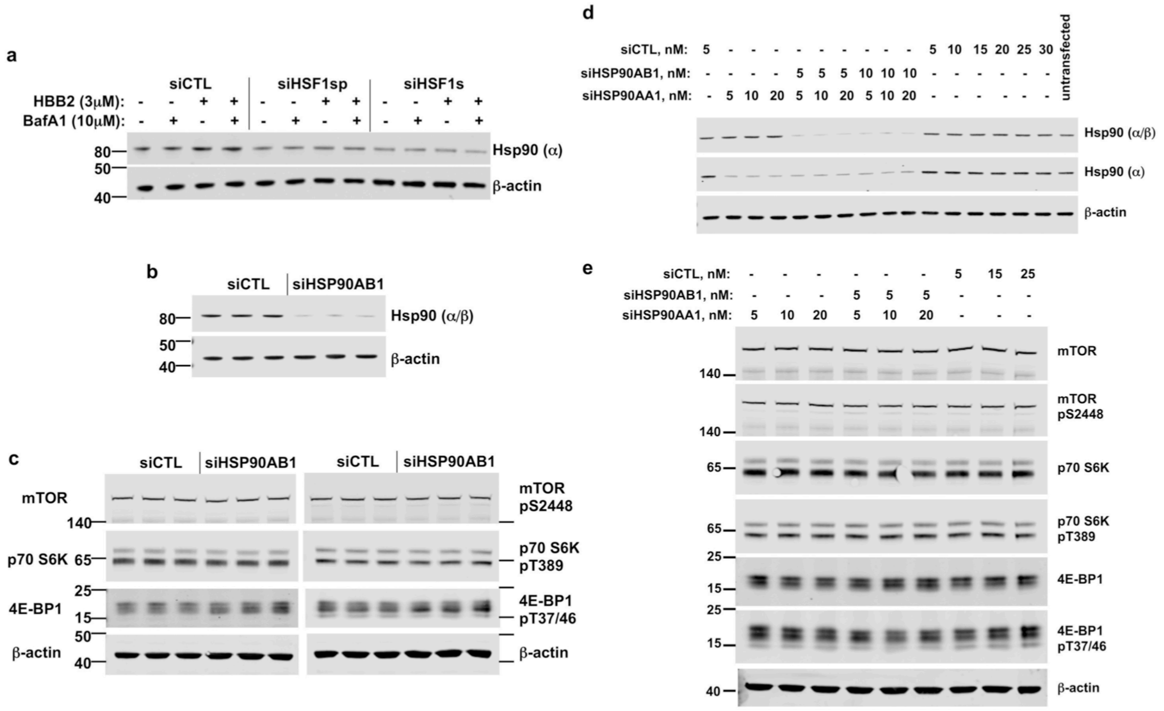

Figure S4. Depletion of Hsp90α and/or Hsp90β does not affect the levels of mTOR and its substrate phosphorylation. Immunoblotting analysis of Hsp90 **(a, b, d)**, mTOR, phosphorylated mTOR (pS2448), p70 S6K, phosphorylated p70 S6K (pT389), 4E-BP1, and phosphorylated 4E-BP1 (pT36/47) **(c, e)** in lysates from U2OS cells, which had been transfected with either control siRNA, HSF1 siRNA, Hsp90α and/or Hsp90β siRNA for 48 h. In **b** and **c**, three separate transfections are shown for each condition. The levels of β-actin served as a loading control.
